# Reversible depressive effect of TNFa on a model of isolated perfused rat heart

**DOI:** 10.1186/cc11940

**Published:** 2013-03-19

**Authors:** BV Nguyen, M Guillouet, MA Giroux-Metges, G Gueret, M Ould-Ahmed, JP Pennec

**Affiliations:** 1 Hôpital d'Instruction des Armées Clermont Tonnerre, Brest, France; 2 Laboratory of Physiology, Faculty of Medicine, Brest, France; 3 University Hospital, Brest, France

## Introduction

Acute myocardial depression in septic shock is common [1]. Myocardial depression is mediated by circulating depressant substances, which until now have been incompletely characterized [2]. The aim of our study was to observe the effects of TNFα on the model of perfused rat heart.

## Methods

After profound anesthesia with pentothal, the Wistar rats were killed by exsanguination. After sternotomy, the heart was taken and connected to the Langendorf column. The apex of the heart was hooked to a strength sensor. Biopac student laboratory software was used to record and analyse heart contractions. Contractions were recorded every 5 minutes during periods of 20 minutes. Control measurements were first recorded. We measured four parameters: heart rate, contraction force, speeds of contraction and relaxation for control, during TNFα (20 ng/ml) exposure and after removal of TNFα. We express the variations of parameters as percentage of the control ± SEM. A paired *t *test was used to compare heart rate, contraction amplitude, speeds of contraction and relaxation with TNFa and control measurements and after removal of TNFα.

## Results

Eight rat hearts Wistar (weight = 325 ± 23 g) were studied. See Table 1.

**Table 1:** 

	TNFα	Removal of TNFα
Heart rate	78 ± 6*	91 ± 5
Contractile	62 ± 8*	91 ± 4
Speed of contraction	72 ± 6*	93 ± 2
Speed of relaxation	53 ± 10*	89 ± 4

Results expressed as percentage of control ± SEM. *P <0.05.

## Conclusion

TNFα decreases significantly the heart rate, contractile force, speeds of contraction and relaxation on isolated perfused rat heart. TNFα probably plays a role in the pathophysiology of cardiomyopathy during septic shock. The partial reversibility of these effects could explain why left ventricular hypokinesia in patients with septic shock is reversible.
